# Integrated Bioinformatic and Experimental Analysis of *SLFN11* Expression and Clinical Significance in Locally Advanced Rectal Cancer

**DOI:** 10.3390/biomedicines14081827

**Published:** 2026-08-13

**Authors:** Jelenko Jelenkovic, Marko Miladinov, Katarina Eric, Katarina Zeljic, Jelena Kotur Stevuljevic, Goran Barisic, Jovana Rosic Stojkovic

**Affiliations:** 1 Clinic for Digestive Surgery–First Surgical Clinic, University Clinical Center of Serbia, 11000 Belgrade, Serbia; jelenkojelenkovic1@gmail.com (J.J.); marko.miladinov90@gmail.com (M.M.); barisic_goran@yahoo.com (G.B.); 2 Department of Pathology, Pathohistology and Medical Cytology, University Clinical Center of Serbia, 11000 Belgrade, Serbia; ketrineric@gmail.com; 3 Faculty of Biology, University of Belgrade, 11000 Belgrade, Serbia; katarina.zeljic@bio.bg.ac.rs; 4 Faculty of Pharmacy, University of Belgrade, 11000 Belgrade, Serbia; jelena.kotur@pharmacy.bg.ac.rs; 5 Faculty of Medicine, University of Belgrade, 11000 Belgrade, Serbia

**Keywords:** Schlafen 11, *SLFN11*, locally advanced rectal cancer, neoadjuvant chemoradiotherapy, gene expression, treatment response

## Abstract

**Background/Objectives**: Schlafen 11 (*SLFN11*) encodes a DNA damage response protein implicated in sensitivity to DNA-damaging therapies and has been proposed as a predictive biomarker in several malignancies. This study aimed to characterize *SLFN11* expression and evaluate its prognostic and predictive significance in locally advanced rectal cancer (LARC). **Methods**: Publicly available datasets were interrogated using UCSC Xena, GEPIA2, TNMplot, KMplot, ROC Plotter, and GEO databases. *SLFN11* expression was assessed by quantitative real-time PCR in paired tumor and adjacent non-tumor tissues collected before and after neoadjuvant chemoradiotherapy (nCRT) from 26 patients with LARC. Associations with clinicopathological characteristics, pathological response, and survival outcomes were analyzed. **Results**: Publicly available datasets demonstrated lower *SLFN11* expression in rectal tumor tissues compared with non-tumor tissues. In our cohort, no significant differences in *SLFN11* expression were observed between paired tumor and adjacent non-tumor tissues either before or after nCRT. However, *SLFN11* expression increased significantly following nCRT in both tissue types. Nevertheless, neither bioinformatic analyses nor our cohort demonstrated a significant association between *SLFN11* expression and survival outcomes. Likewise, no significant predictive value of *SLFN11* expression for response to nCRT was observed in ROC Plotter analysis, most GEO datasets, or our clinical cohort. The prognostic and predictive findings should be considered exploratory due to limited statistical power. **Conclusions**: Although *SLFN11* expression is reduced in rectal tumor tissues and appears to be influenced by nCRT, its prognostic and predictive value in LARC remains uncertain. Larger studies are warranted to clarify its biological and clinical significance.

## 1. Introduction

Colorectal cancer (CRC) represents a significant global cancer burden, ranking third in incidence and second in cancer-related mortality worldwide [1]. According to recent global estimates, CRC accounts for approximately 9.6% of all cancer cases and 9.3% of cancer-related deaths, with more than 1.9 million new diagnoses and over 900,000 deaths reported worldwide in 2022 alone [1]. Rectal cancer accounts for nearly 40% of both new cases and related deaths, underscoring its considerable clinical and public health impact [2]. Locally advanced rectal cancer (LARC) is defined as a tumor extending beyond the rectal wall and/or involving regional lymph nodes, in the absence of distant metastasis. Despite substantial advances in multimodal treatment, LARC remains a major clinical challenge, particularly with respect to prognosis and prediction of therapeutic response. Surgical resection represents the cornerstone of curative treatment for rectal cancer; however, standard management of LARC includes preoperative (neoadjuvant) chemoradiotherapy (nCRT), with the aim of tumor downstaging and downsizing, frequently followed by adjuvant chemotherapy to reduce the risk of distant relapse [3,4]. More recently, total neoadjuvant therapy (TNT) has emerged as an alternative strategy that incorporates all systemic therapy into the preoperative setting [5,6].

Response to nCRT is highly heterogeneous and difficult to predict. A complete pathological response is achieved in only 10–30% of patients, while the majority demonstrate partial response or resistance to therapy [7,8]. Consequently, there is a pressing need to improve our understanding of the molecular mechanisms underlying LARC in order to identify robust prognostic and predictive biomarkers. Such biomarkers could enable better stratification of patients according to expected treatment benefit, thereby optimizing therapeutic decision-making and minimizing unnecessary treatment-related toxicity and preserving quality of life.

*SLFN11* (Schlafen family member 11) gene encodes a protein with putative DNA/RNA helicase and endonuclease functions. Under replication stress, SLFN11 acts as an S-phase checkpoint regulator by binding to replication fork and inducing irreversible replication blockage through chromatin opening near initiation sites. This ultimately triggers cell death [9,10,11,12]. It has gained prominence in cancer biology because of its ability to sensitize tumor cells to DNA-damaging chemotherapeutic agents (DDAs), including platinum compounds, topoisomerase inhibitors, and DNA synthesis inhibitors, as well as to targeted poly (ADP-ribose) polymerase (PARP) inhibitors [13,14,15]. Since SLFN11 exerts its effects by selectively blocking replication in response to DNA damage, ultimately leading to cell death in cancer cells, its expression has been associated with improved responses to therapies targeting DNA repair pathways, making it a promising target for precision oncology [16]. *SLFN11* is broadly expressed across various cancer types and has recently been identified as a biomarker predicting response to a wide range of DDAs and PARP inhibitors across multiple cancer types, including small cell lung cancer, sarcoma, breast cancer, ovarian cancer, CRC, gastric cancer, and mesothelioma in preclinical settings [17]. To the best of our knowledge, there are insufficient data in the literature to support the role of *SLFN11* as a prognostic or predictive biomarker in LARC. We hypothesized that *SLFN11* expression would be upregulated following nCRT and that higher expression would be associated with a more favorable response to treatment. Therefore, this study aimed to comprehensively evaluate *SLFN11* mRNA expression in LARC by integrating bioinformatic analyses with clinical gene expression data and to assess its prognostic and predictive significance.

## 2. Materials and Methods

### 2.1. Bioinformatic Analysis of Publicly Available Databases and Web-Based Platforms

*SLFN11* expression levels were analyzed in tumor and non-tumor tissues from rectal cancer patients using data from the Rectal Adenocarcinoma (READ) project of the Genomic Data Commons–The Cancer Genome Atlas (GDC–TCGA). The GDC–TCGA READ dataset was retrieved and analyzed through the UCSC Xena online platform (University of California, Santa Cruz Xena; http://xena.ucsc.edu; accessed 12 December 2025 and 14 January 2026) [18]. From the GDC–TCGA READ cohort, 177 samples with available *SLFN11* gene expression data were first identified. These were subsequently restricted to Primary Tumor samples (tumor tissue) and Solid Tissue Normal samples (non-tumor tissue). Cases were then limited to those diagnosed with American Joint Committee on Cancer (AJCC) staging system pathologic stages II and III at initial diagnosis. Further refinement included tumors with a primary site of rectum or rectosigmoid junction and a disease type classified as adenoma or adenocarcinoma according to TCGA annotations. Following this stepwise selection process (Supplementary Figure S1), 89 samples were retained for analysis, comprising 86 tumor and 3 non-tumor tissue samples. The three non-tumor tissue samples were matched to corresponding tumor samples within the dataset. The finalized dataset was downloaded and used for subsequent statistical analyses.

Comparisons of *SLFN11* expression between tumor and non-tumor tissues in rectal cancer, as well as survival analyses based on high and low *SLFN11* expression in rectal tissues, were performed using GEPIA2 (http://gepia2.cancer-pku.cn/#index; accessed on 16 January 2026) [19]. GEPIA2 integrates RNA sequencing data from the TCGA and GTEx projects. For rectal cancer-specific analyses, data from the TCGA READ (Rectal Adenocarcinoma) cohort were used, comprising 92 rectal adenocarcinoma samples and 10 paired peritumor normal tissue samples. In addition, tumor samples were compared with a larger reference set of 308 normal colon tissue samples derived from the GTEx project.

*SLFN11* expression was compared between paired and unpaired tumor and non-tumor tissues from rectal cancer patients using the TNMplot web platform (https://tnmplot.com/analysis; accessed on 11 December 2025 and 16 January 2026) [20]. The dataset included 9 patients with paired tumor–non-tumor samples, as well as 166 tumor samples and 243 normal tissue samples with available *SLFN11* RNA-sequencing expression data.

The prognostic significance of *SLFN11* was evaluated using the Kaplan–Meier Plotter (KMplot) platform (https://kmplot.com/analysis/; accessed on 16 January 2026) based on RNA sequencing data [21]. The analysis was limited to stage II–III rectal adenocarcinoma patients of White ethnicity to ensure comparability with the study cohort, resulting in 51 patients included. Patients were stratified into high and low *SLFN11* expression groups using the median tumor expression level, and survival differences were assessed by Kaplan–Meier analysis with log-rank testing, with hazard ratios and 95% confidence intervals calculated.

The predictive significance of *SLFN11* in rectal cancer with respect to response to chemotherapy and radiotherapy was evaluated using the ROC Plotter web tool (https://rocplot.com/; accessed on 27 January 2026) with JetSet settings [22]. A total of 98 rectal cancer patients who received both fluoropyrimidine-based chemotherapy and radiotherapy were available on this web platform for analysis, of whom 34 were responders and 64 were non-responders according to RECIST criteria. Classifier performance was assessed using the area under the receiver operating characteristic curve (AUC), while the optimal *SLFN11* expression cut-off for discriminating between responders and non-responders was determined using the platform’s default algorithm. Detailed clinicopathological information, including disease stage and specific treatment protocols, was not available within the platform.

To further investigate *SLFN11* expression in relation to response to neoadjuvant chemoradiotherapy (nCRT), the Gene Expression Omnibus (GEO) database (https://www.ncbi.nlm.nih.gov/geo/; accessed on 28 January 2026) was queried for relevant datasets [23]. The search was performed using the keywords “LARC” OR “locally advanced rectal cancer” AND “Homo sapiens”, with all available expression profiling study types included. The initial search yielded 39 datasets. Following manual review of the study summaries, 16 datasets were examined in greater detail, of which 8 were identified as relevant and contained both *SLFN11* expression data and treatment response information (GSE94104, GSE68204 (GPL6480 platform), GSE145666, GSE53781, GSE242786, GSE46862, GSE150082, GSE145037). Differential expression analysis of the selected datasets was performed using GEO2R (https://www.ncbi.nlm.nih.gov/geo/geo2r/; accessed on 28 and 29 January 2026) with default settings. Statistical significance was defined as *p* < 0.05, with a log2 fold-change threshold of 0.

### 2.2. Patients and Tissue Collection

This study included 26 patients with LARC (T3/T4, N0, M0 or any T, N1/N2, M0) treated between April 2019 and October 2020 at the Clinic for Digestive Surgery–First Surgical Clinic, University Clinical Center of Serbia, Belgrade. Inclusion criteria comprised patients with histopathologically confirmed rectal adenocarcinoma and radiologically confirmed LARC who underwent long-course nCRT followed by total mesorectal excision. Exclusion criteria included stage I or IV disease at presentation, histopathologically confirmed mucinous rectal adenocarcinoma on pretreatment biopsy, synchronous colorectal cancer or other malignancies, an American Society of Anesthesiologists score > 3, and lack of written informed consent.

A total of 104 tissue samples were collected from the 26 included patients. Pretreatment tumor and adjacent non-tumor rectal tissue samples were obtained by biopsy prior to therapy using anoscopy or rectoscopy, depending on tumor location. Tumors were classified as located in the lower rectum (≤5 cm from the anal verge) or mid-rectum (6–10 cm from the anal verge). All biopsy specimens were histopathologically reviewed to confirm rectal adenocarcinoma, and each sample was divided to allow pathological verification of tumor tissue and adjacent normal mucosa.

Pretherapeutic clinical TNM (cTNM) staging was determined using magnetic resonance imaging (MRI), which was also used to assess the distance to the circumferential resection margin (CRM) and the presence of extramural vascular invasion (EMVI).

All patients subsequently received nCRT, consisting of a total radiation dose of 50.4 Gray administered in 28 fractions, combined with two cycles of chemotherapy administered during the first and fifth weeks of radiotherapy. The chemotherapy regimen comprised 5-fluorouracil (5-FU) at a dose of 425 mg/m^2^ and leucovorin at 20 mg/m^2^.

Surgical resection was performed 8–12 weeks after completion of nCRT. Following surgery, tumor and adjacent non-tumor tissue samples were collected again from the resected bowel specimen immediately after excision. Pathological TNM (pTNM) staging was determined by an experienced pathologist according to the 8th edition of the AJCC staging system (2017) [24].

Tumor response to nCRT was evaluated pathologically using the Mandard tumor regression grade (TRG) system. Patients were classified as responders (TRG 1–2) or non-responders (TRG 3–4) based on histopathological assessment of the resected specimen.

All tissue samples were placed in TRIzol™ Reagent (Invitrogen, Carlsbad, CA, USA) immediately after collection and stored at −80 °C until further analysis.

Follow-up was conducted at 3-month intervals in the first postoperative year, followed by 6-month intervals during the second year and annual assessments thereafter, with the most recent follow-up performed in September 2024. The median follow-up was 57 months.

### 2.3. Ethics Statement

Ethical approval for this study was obtained from the Ethics Committee of the Faculty of Medicine, University of Belgrade (approval number 1550/V-2, 31 May 2019) and the Ethics Committee of the University Clinical Center of Serbia (approval number 455/24, 7 May 2026). The study was conducted in accordance with the Declaration of Helsinki. All participants provided written informed consent prior to enrollment in the study.

### 2.4. RNA Extraction and cDNA Synthesis

Total RNA was extracted from collected tissue samples using TRIzol™ Reagent (Invitrogen, Carlsbad, CA, USA) according to the manufacturer’s protocol. RNA concentration and purity were assessed using an Ultrospec 3300 Pro spectrophotometer (Amersham Biosciences, Little Chalfont, UK).

Complementary DNA (cDNA) was synthesized from 20 ng of total RNA in a 20 μL reaction volume using the High-Capacity cDNA Reverse Transcription Kit (Applied Biosystems™, Foster City, CA, USA), following the manufacturer’s instructions. Each reaction contained 10× RT buffer, 25× dNTP mix (100 mM), 10× random primers, MultiScribe™ reverse transcriptase, and RNase inhibitor. Reverse transcription was performed under the following conditions: 25 °C for 10 min, 37 °C for 120 min, and 85 °C for 5 min.

### 2.5. qPCR

Real-time quantitative polymerase chain reaction (qPCR) was performed to measure the relative expression of *SLFN11* using a TaqMan™ Gene Expression Assay (Thermo Fisher Scientific, Waltham, MA, USA; assay ID: Hs00536981_m1). Beta-actin (*ACTB*, assay ID: Hs99999903_m1) was used as endogenous control gene for normalization.

qPCR reactions were carried out under the following cycling conditions: 95 °C for 10 min, followed by 40 cycles of 95 °C for 15 s and 60 °C for 1 min. All qPCR reactions were performed in technical duplicates, and the mean Ct value of the two replicates was used for subsequent ΔCt calculations. Reactions with a Ct difference greater than 0.5 between technical duplicates were repeated to ensure reproducibility. Relative gene expression levels were determined using the 2^−ΔCt^ method [25].

### 2.6. Statistical Analysis

Statistical analyses were performed using GraphPad Prism version 8.4.3 (GraphPad Software Inc., San Diego, CA, USA). The normality of data distribution was assessed using the Shapiro–Wilk test. Since the data did not follow a normal distribution, nonparametric statistical methods were applied. Relative gene expression data are presented as median and interquartile range (IQR) of 2^−ΔCt^ values, or as mean ± standard deviation (SD) for publicly available datasets. Differences between two independent groups were analyzed using the Mann–Whitney U test, whereas paired samples were compared using the Wilcoxon matched-pairs signed-rank test. Correlations between continuous variables were assessed using Spearman’s rank correlation coefficient (rho). Corrections for multiple comparisons were performed using the Benjamini–Hochberg false discovery rate method. Overall survival and disease-free survival were evaluated using the Kaplan–Meier method, and differences between survival curves were assessed using the log-rank test. For survival analyses, patients were stratified into high- and low-expression groups according to the median *SLFN11* expression value. The predictive performance of *SLFN11* expression for response to nCRT was evaluated using receiver operating characteristic (ROC) curve analysis, with the area under the curve (AUC) and its 95% confidence interval (95% CI) calculated. The optimal cut-off value was determined using the maximum Youden index, and the corresponding sensitivity and specificity were reported. Hazard ratio (HR) with 95% CI was calculated using Cox proportional regression analysis in IBM SPSS Statistics, version 22.00 (IBM Corp., Armonk, NY, USA). The statistical power of the study was calculated using G*Power, version 3.1.9.7 (University of Kiel, Kiel, Germany).

All statistical tests were two-sided, and *p* values < 0.05 were considered statistically significant.

## 3. Results

### 3.1. Bioinformatic Analysis of SLFN11 Using Publicly Available Databases and Web-Based Platforms

**Figure 1:**
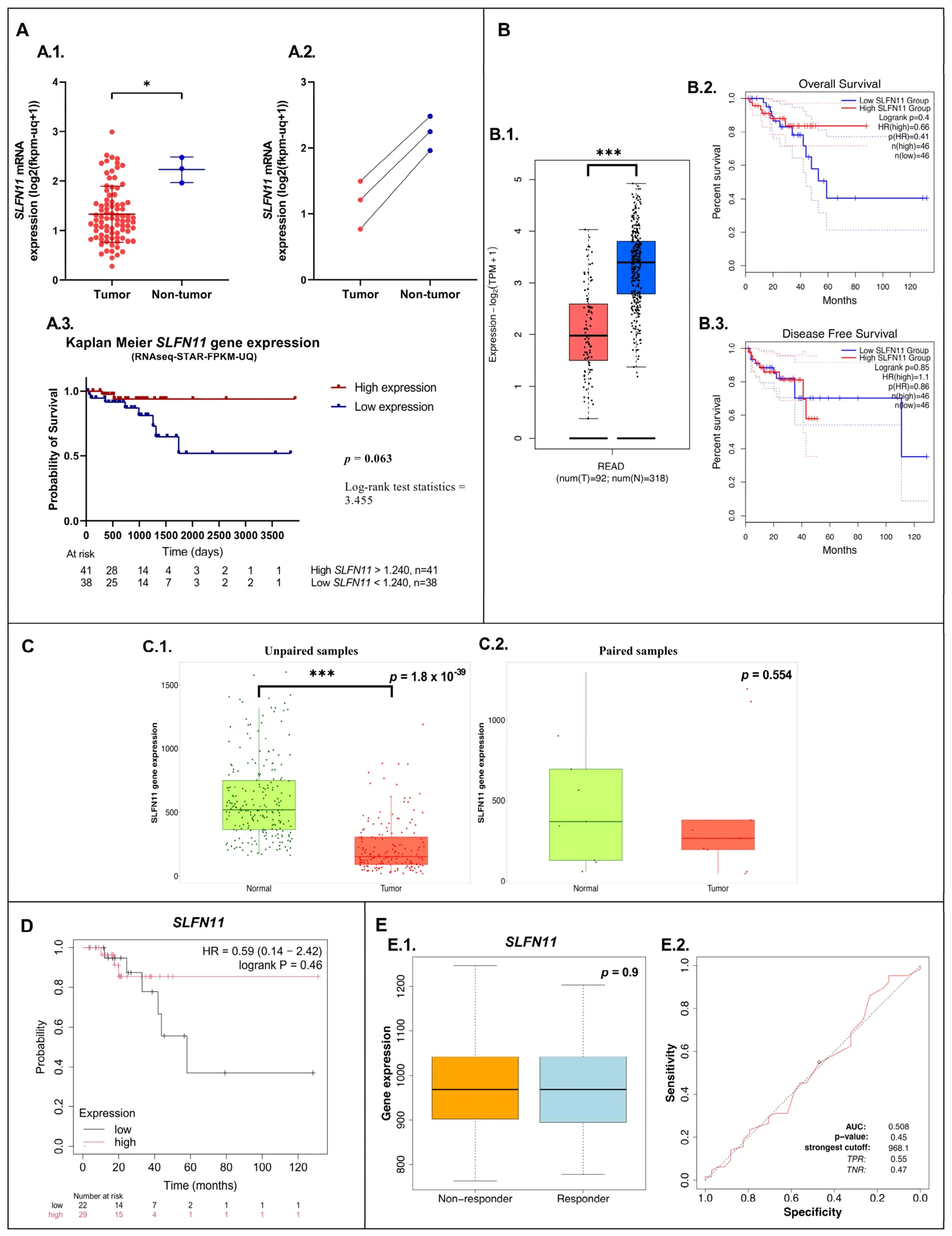
Analysis of *SLFN11* expression and its clinical significance in publicly available rectal cancer datasets. (**A**) UCSC Xena Browser (GDC–TCGA READ dataset): (**A.1.**) comparison of *SLFN11* expression between tumor and non-tumor tissues, (**A.2.**) paired analysis of matched tumor and adjacent non-tumor tissues, and (**A.3.**) overall survival according to *SLFN11* expression levels. Data are presented as mean ± standard deviation (SD). (**B**) GEPIA2 analysis: (**B.1.**) *SLFN11* expression in rectal adenocarcinoma and non-tumor colorectal tissues, (**B.2.**) overall survival, and (**B.3**.) disease-free survival according to *SLFN11* expression. (**C**) TNMplot analysis of *SLFN11* expression in (**C.1.**) unpaired and (**C.2.**) paired tumor and non-tumor rectal tissues. (**D**) Kaplan–Meier overall survival analysis based on *SLFN11* expression using the KMplot platform. (**E**) ROC Plotter analysis of the predictive value of *SLFN11* expression for response to fluoropyrimidine-based chemoradiotherapy in rectal cancer, including (**E.1.**) comparison of expression levels between responders and non-responders and (**E.2.**) ROC curve analysis. *p* values are shown within individual panels. * *p* < 0.05, *** *p* < 0.001. Graphs generated by GEPIA2, TNMplot, KMplot, and ROC Plotter are presented in their original format (with asterisks added to indicate statistical significance), as raw data are not available for download from these platforms.

*SLFN11* expression levels were analyzed in tumor and non-tumor tissues from patients with stage II and III rectal cancer using data from the GDC–TCGA READ project accessed via UCSC Xena. *SLFN11* expression was significantly lower in rectal tumor tissue compared with non-tumor tissue (Mann–Whitney U test, *p* = 0.011; Figure 1(A.1.)). In the subset of paired samples (*n* = 3), no significant difference in *SLFN11* expression was observed between tumor and matched non-tumor tissues from the same patients (Wilcoxon matched-pairs signed-rank test, *p* = 0.250; Figure 1(A.2.)). Additionally, overall survival was evaluated in patients with available tumor *SLFN11* expression and survival data (*n* = 79). Patients were stratified into high and low *SLFN11* expression groups, and no significant difference in overall survival was observed between the groups (*p* = 0.063; Figure 1(A.3.)).

Using the GEPIA2 web server, *SLFN11* expression was found to be significantly downregulated in rectal adenocarcinoma tissues compared with non-tumor tissues (*p* < 0.001; Figure 1(B.1.)). When compared with the 10 paired peritumoral normal rectal tissue samples, *SLFN11* expression showed a trend toward downregulation in the 92 rectal adenocarcinoma samples, although the difference was not statistically significant (*p* > 0.05). No significant differences in overall or disease-free survival were observed when patients were stratified into high and low *SLFN11* expression groups based on *SLFN11* expression levels in rectal adenocarcinoma tissue (*p* = 0.4, *p* = 0.85, respectively; Figure 1(B.2.,B.3.)).

TNMplot analysis of *SLFN11* differential expression between unpaired tumor and non-tumor rectal cancer tissues revealed significant downregulation of *SLFN11* in tumor tissue compared with normal tissue (*p* = 1.8 × 10^−39^). In contrast, although a similar trend was observed in paired tumor–non-tumor samples, the difference was not statistically significant (*p* = 0.554; Figure 1C).

To assess the prognostic value of *SLFN11*, the KMplot web platform was used. No significant differences in survival were observed between rectal adenocarcinoma patients with high versus low *SLFN11* expression (*p* = 0.46, log-rank test; Figure 1D).

Analysis using the ROC Plotter web platform showed no predictive value of *SLFN11* for response to fluoropyrimidine-based chemotherapy and radiotherapy in rectal cancer (*p* = 0.9, Mann–Whitney U test; Figure 1(E.1.,E.2.)).

**Figure 2:**
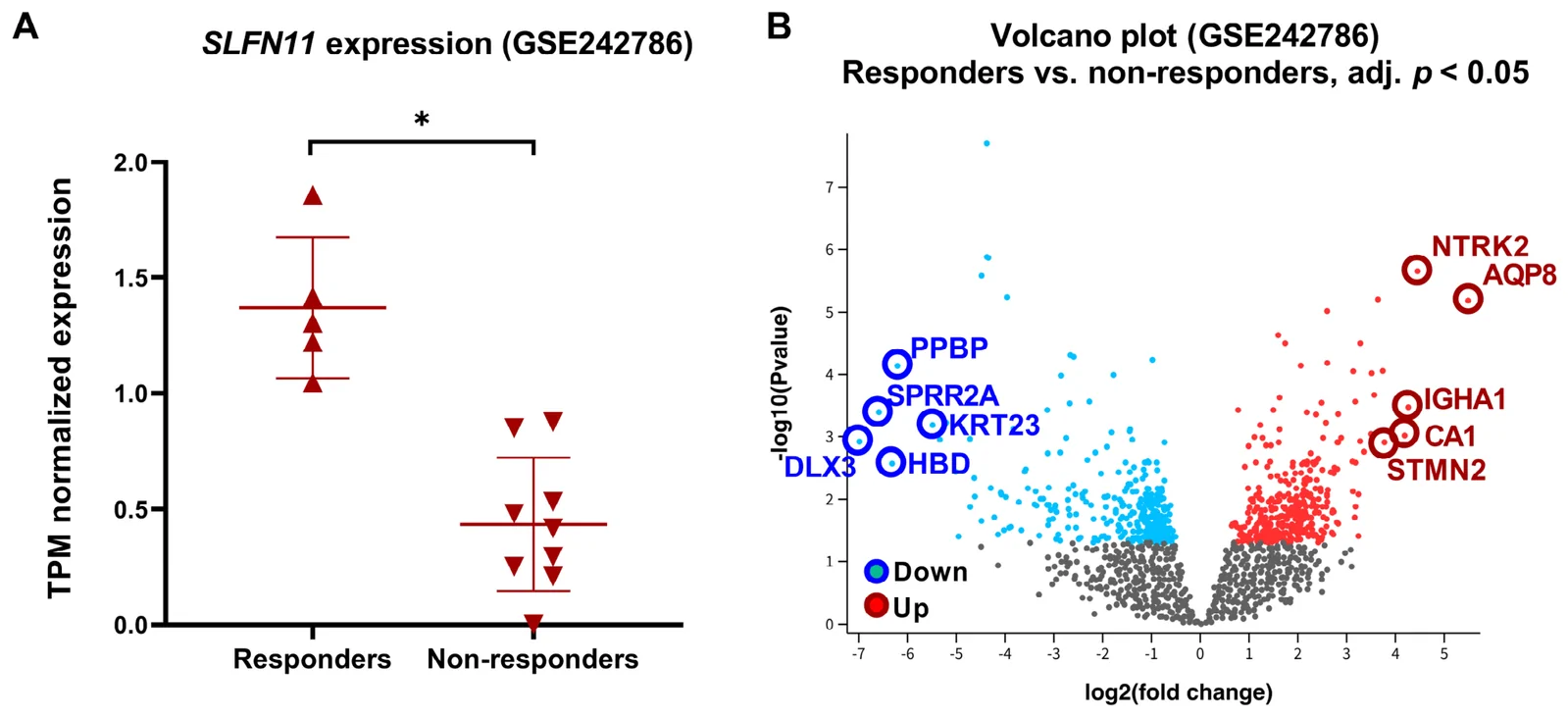
(**A**) Comparison of transcripts per million (TPM)-normalized *SLFN11* expression levels between patients with complete and incomplete response to neoadjuvant chemoradiotherapy; expression values were downloaded from the GEO platform for visualization. (**B**) Volcano plot illustrating differential gene expression between responders and non-responders in the GSE242786 dataset, generated using GEO2R. The top five upregulated and top five downregulated genes were selected based on adjusted *p*-value and log2 fold-change values and are indicated on the plot. Gray points represent genes that were not significantly differentially expressed between responders and non-responders. * Adjusted (adj.) *p* < 0.05.

Across the GEO datasets analyzed, *SLFN11* expression did not differ significantly between good and poor responders in 7 of the 8 datasets (GSE94104, GSE68204, GSE145666, GSE53781, GSE46862, GSE150082, GSE145037), as summarized in Table 1. In contrast, dataset GSE242786 showed a significant difference in *SLFN11* expression between response groups, with significantly lower expression in patients with an incomplete response compared with those achieving a complete response (adjusted *p* = 0.04; Figure 2).

### 3.2. SLFN11 Expression in the LARC Patient Cohort and Its Association with Clinicopathological Characteristics, Survival Outcomes and Treatment Response

Demographic and clinicopathological characteristics of the 26 LARC patients included in the study are presented in Table 2. We investigated the association between *SLFN11* expression in tumor tissues collected before and after nCRT and demographic as well as clinicopathological characteristics. The results are summarized in Table 2. Pre-treatment *SLFN11* expression was significantly positively correlated with CEA levels after nCRT (rho = 0.431, *p* = 0.028, Spearman’s correlation), but the significance was lost after correction for multiple comparisons (adjusted *p* = 0.121, Benjamini–Hochberg false discovery rate). Post-treatment tumor *SLFN11* expression was significantly associated with disease outcome (*p* = 0.028, Mann–Whitney U test), but this association did not remain significant after correction for multiple comparisons (adjusted *p* = 0.336, Benjamini–Hochberg false discovery rate). Therefore, these findings were considered exploratory. No other significant associations or correlations were observed between *SLFN11* expression before or after nCRT and the evaluated demographic or clinicopathological characteristics.

**Figure 3:**
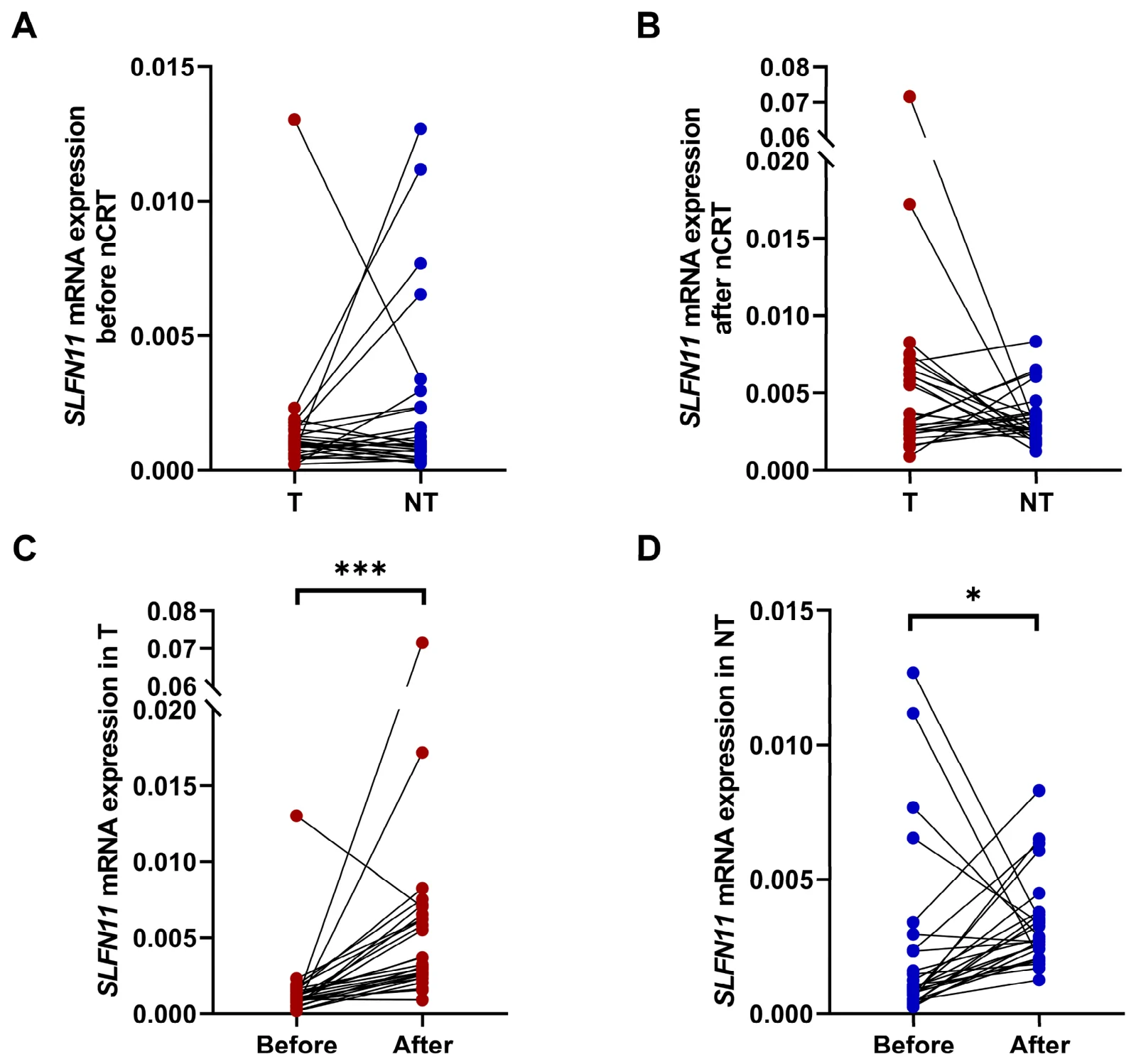
*SLFN11* expression in paired tumor (T) and adjacent non-tumor (NT) tissues collected before and after neoadjuvant chemoradiotherapy (nCRT). Comparison of *SLFN11* expression (**A**) between tumor and matched adjacent non-tumor tissues before nCRT; (**B**) between tumor and matched adjacent non-tumor tissues after nCRT; (**C**) in paired tumor tissues collected before and after nCRT, showing increased expression following treatment (*p* < 0.001); and (**D**) in paired adjacent non-tumor tissues collected before and after nCRT, showing increased expression following treatment (*p* = 0.029). The y-axis break is indicated by a discontinuity mark (\\) in figures (**B**,**C**). *p* values were calculated using the Wilcoxon matched-pairs signed-rank test. * *p* < 0.05, *** *p* < 0.001.

Comparison of paired tumor and adjacent non-tumor tissues collected from each patient before and after nCRT revealed no significant differences in *SLFN11* expression between tumor and non-tumor tissues either before or after nCRT (Figure 3A,B; Wilcoxon matched-pairs signed-rank test, *p* = 0.291 and *p* = 0.227, respectively).

When paired tumor tissues obtained from the same patients before and after nCRT were compared, *SLFN11* expression was significantly higher in post-treatment tumor tissues (Figure 3C; Wilcoxon matched-pairs signed-rank test, *p* < 0.001). Similarly, *SLFN11* expression was significantly increased in adjacent non-tumor tissues collected after nCRT compared with matched pre-treatment non-tumor samples (Figure 3D; Wilcoxon matched-pairs signed-rank test, *p* = 0.029).

**Figure 4:**
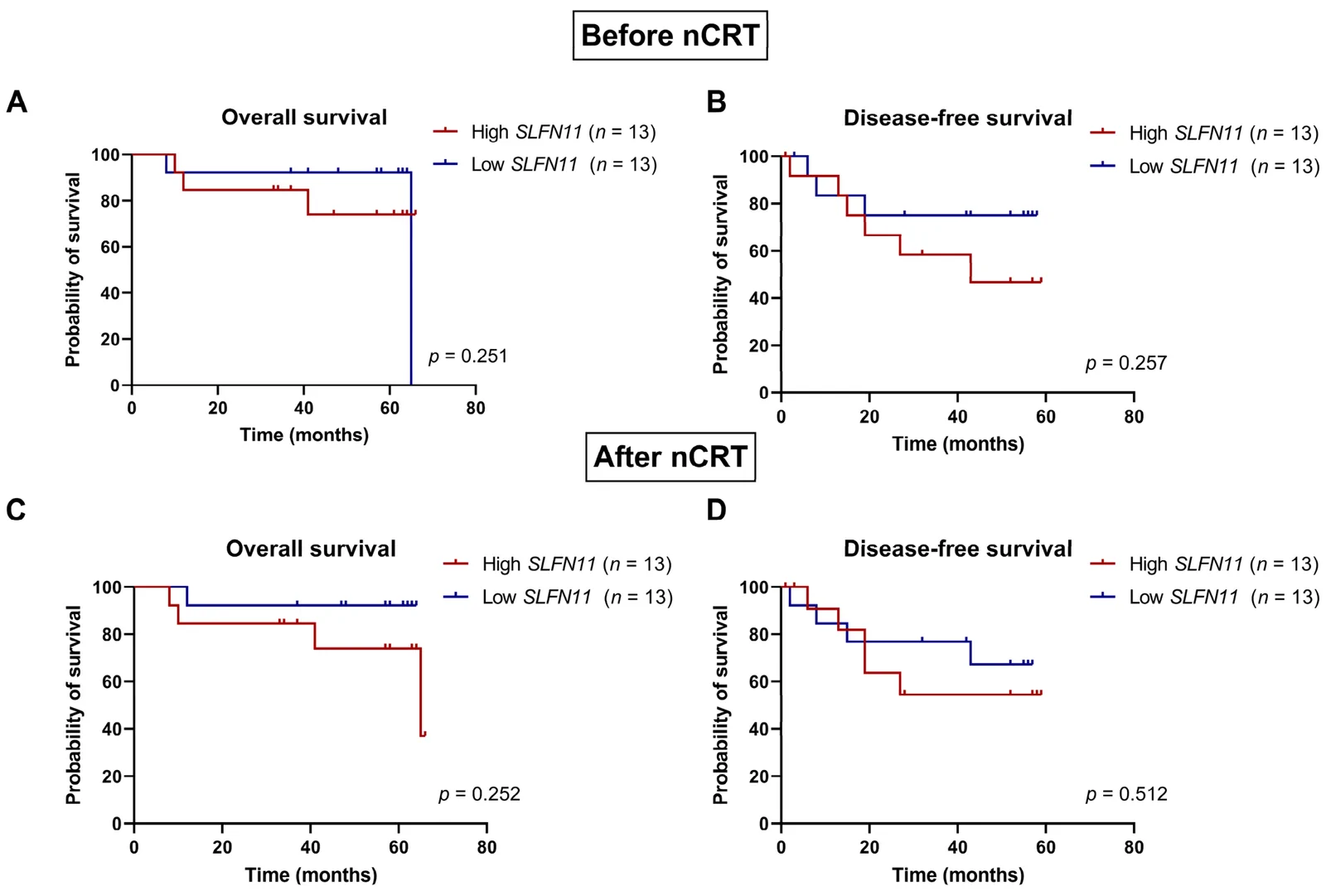
Kaplan–Meier survival analysis according to *SLFN11* expression in tumor tissues from patients with locally advanced rectal cancer. Overall survival stratified by *SLFN11* expression (**A**) before and (**C**) after neoadjuvant chemoradiotherapy (nCRT), and disease-free survival stratified by *SLFN11* expression (**B**) before and (**D**) after nCRT. Patients were categorized into high- and low-expression groups using the median expression (2^−ΔCt^) value as the cutoff. Log-rank test *p* values are shown within the panels.

To assess the prognostic significance of *SLFN11* expression, patients were stratified into low- and high-expression groups using the median expression value as the cutoff. Kaplan–Meier survival analysis showed no significant differences in overall survival between patients with high and low *SLFN11* expression in tumor tissues collected either before nCRT (*p* = 0.251) or after nCRT (*p* = 0.252) (Figure 4A,C). Similarly, *SLFN11* expression levels were not associated with disease-free survival, regardless of whether expression was evaluated in pre-treatment or post-treatment tumor samples (Figure 4B,D; *p* = 0.257 and *p* = 0.512, respectively).

Cox regression analysis demonstrated no statistically significant association between *SLFN11* expression and death or recurrence in patients with LARC (Table 3). Given the limited number of events, these findings should be taken with caution.

**Figure 5:**
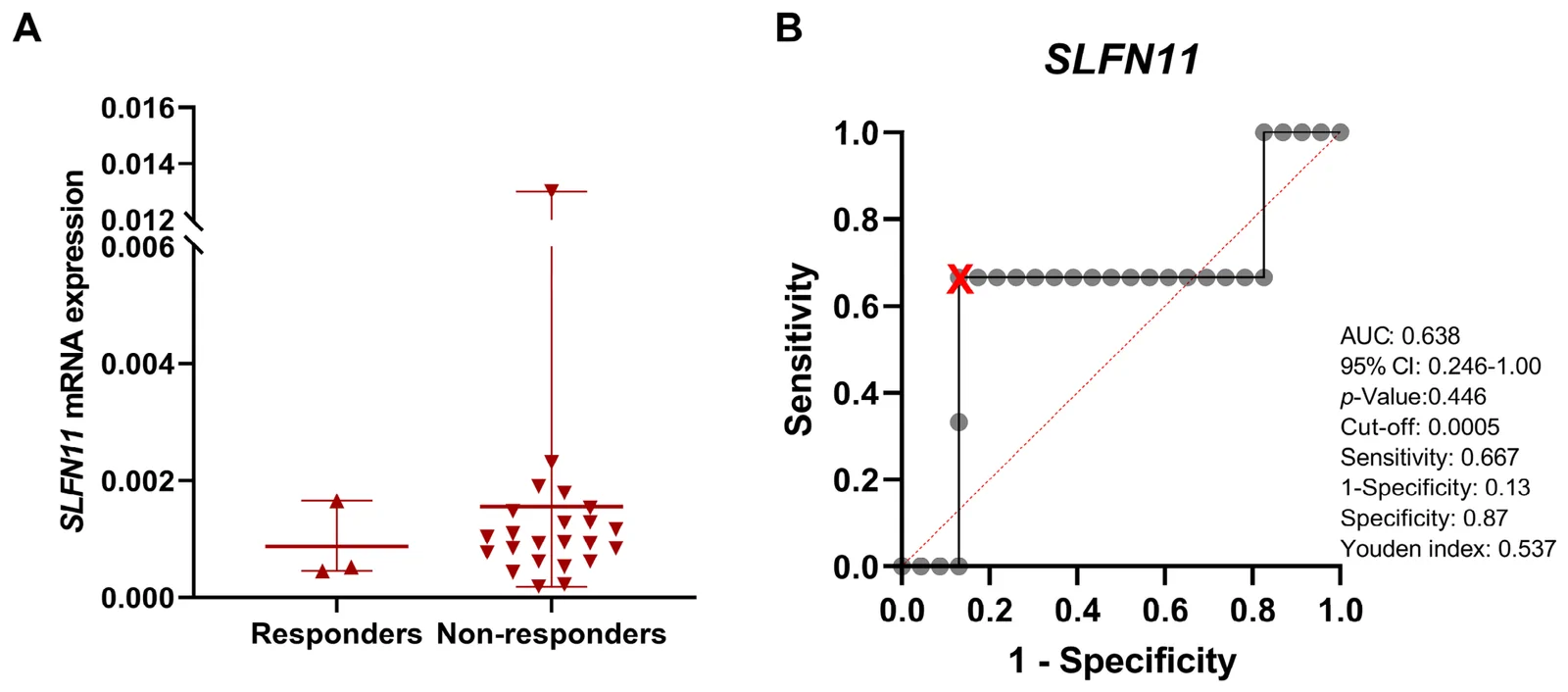
Predictive value of pre-treatment *SLFN11* expression for response to neoadjuvant chemoradiotherapy in patients with locally advanced rectal cancer. (**A**) Comparison of *SLFN11* expression between responders (Mandard TRG 1–2) and non-responders (Mandard TRG 3–4). (**B**) ROC curve analysis of pre-treatment *SLFN11* expression for discrimination between responders and non-responders. The y-axis break is indicated by a discontinuity mark (\\) in figure (**A**). In figure (**B**), the red “×” indicates the ROC point corresponding to the selected cut-off value, while the red diagonal dotted line represents the reference line for a classifier with no discriminatory ability (AUC = 0.5).

To evaluate the predictive significance of *SLFN11* expression, pre-treatment tumor tissue expression levels were compared between responders (Mandard TRG 1–2) and non-responders (Mandard TRG 3–4) to nCRT. No significant difference in *SLFN11* expression was observed between the two response groups (Figure 5A; Mann–Whitney U test, *p* = 0.490). ROC curve analysis demonstrated no significant predictive value of pre-treatment *SLFN11* expression for pathological response to nCRT (AUC = 0.638, 95% CI = 0.246–1.000, *p* = 0.446; Figure 5B).

## 4. Discussion

SLFN11 has emerged as a promising biomarker of sensitivity to DNA-damaging agents across multiple malignancies due to its ability to enhance replication stress and promote irreversible cell death following DNA damage [16,32]. Consequently, increased *SLFN11* expression has frequently been associated with enhanced sensitivity to DNA-damaging anticancer therapies, particularly platinum compounds, topoisomerase inhibitors, and PARP inhibitors [13,17,33]. Emerging evidence further suggests that *SLFN11* may contribute to tumor radiosensitivity, although this association has been investigated less extensively [34]. However, the role of *SLFN11* in rectal cancer remains insufficiently characterized, and data regarding its expression patterns and clinical significance are limited. In the present study, we performed an integrated bioinformatic analysis and gene expression analysis of clinical tissue samples to investigate *SLFN11* expression patterns and evaluate its prognostic and predictive significance in LARC patients treated with fluoropyrimidine-based neoadjuvant chemoradiotherapy.

Analysis of publicly available datasets (UCSC Xena, GEPIA2, TNMPlot) demonstrated lower *SLFN11* expression in rectal tumor tissues compared with non-tumor tissues in unpaired samples. In contrast, paired analyses were inconclusive due to limited number of paired samples and insufficient statistical power, particularly in the UCSC Xena TCGA cohort (1 − β = 0.11, effect size = 0.8, α = 0.05). This limited number of eligible normal tissues highlights the scarcity of well-characterized matched datasets in LARC. Therefore, this comparison was not considered standalone evidence but rather a complementary observation evaluated together with results from our clinical cohort, warranting further validation in larger cohorts. In our clinical cohort, no significant differences in *SLFN11* expression were observed between paired tumor and adjacent non-tumor tissues either before or after nCRT. The integration of multiple independent public resources together with a clinically characterized patient cohort represents a strength of the present study, as it enabled a comprehensive evaluation of *SLFN11* across different analytical platforms. Although some findings were not fully consistent, these discrepancies likely reflect methodological differences between datasets, including tissue sources, sample size, gene expression platforms, data normalization procedures, and bioinformatic analysis pipelines.

In our clinical cohort, *SLFN11* expression increased significantly following treatment in both tumor and non-tumor tissues, suggesting that exposure to chemoradiotherapy may influence *SLFN11* transcriptional regulation in rectal tissues. Although non-tumor tissue samples were collected approximately 8 cm from the tumor margin, outside the region expected to receive the strongest local radiation-induced effects, the contribution of a broader treatment-related field effect cannot be excluded, and the observed increase in *SLFN11* expression may therefore, at least in part, reflect a treatment-induced DNA damage response to nCRT rather than a tumor-specific phenomenon.

Consistent with our findings, Zoppoli et al. reported substantial variability in *SLFN11* expression across colorectal and ovarian cancers and lower mean *SLFN11* expression in tumor tissues than in corresponding normal tissues in datasets retrieved from The Cancer Genome Atlas [16]. Following nCRT, this trend was reversed in our cohort, with higher *SLFN11* expression observed in tumor tissues than in adjacent non-tumor tissues, albeit without statistical significance. Interestingly, *SLFN11* expression increased significantly following nCRT in both tumor and adjacent non-tumor tissues, suggesting that exposure to chemoradiotherapy may induce *SLFN11* expression as part of the cellular response to treatment-induced DNA damage. This observation is consistent with the established role of SLFN11 in DNA damage response pathways and warrants further investigation into the molecular and epigenetic mechanisms regulating *SLFN11* expression following nCRT. Notably, the increase appeared more pronounced in tumor tissues, which may reflect differential biological responses of malignant and non-malignant tissues to treatment. Tang et al. reported that *SLFN11* overexpression sensitized HCT116 colorectal cancer cells to several DNA-damaging agents, including topoisomerase inhibitors and cisplatin, supporting its potential role as a predictive biomarker of treatment response [35]. In the same study, class I histone deacetylase (HDAC) inhibitors were shown to induce *SLFN11* expression in fibrosarcoma cell lines, patients with cutaneous T-cell lymphoma, and circulating malignant T cells, both *in vitro* and *in vivo*, providing mechanistic evidence that epigenetic silencing of *SLFN11* contributes to resistance to DNA-damaging therapies and may be partially reversed by HDAC inhibition [35]. Epigenetic regulation of *SLFN11* has also been linked to resistance to platinum-based chemotherapy through promoter CpG island hypermethylation, which has been described in both cancer cell lines and patients with ovarian and non-small cell lung cancer [36]. Importantly, *SLFN11* promoter hypermethylation was detected in all colorectal cancer cell lines included in the NCI-60 panel [36], suggesting that epigenetic silencing may represent one mechanism of *SLFN11* inactivation in at least a subset of colorectal cancers. Consistent with these findings, He et al. demonstrated that promoter methylation-mediated silencing of *SLFN11* contributes to colorectal cancer progression, reduced sensitivity to cisplatin, and poorer clinical outcomes [37]. Moreover, *SLFN11* methylation was observed in more than half of colorectal cancer samples but was absent in non-neoplastic colorectal mucosa, and was significantly associated with older age, worse 5-year overall survival, and shorter relapse-free survival [37]. In agreement with these observations, Takashima et al. reported consistently negative SLFN11 immunohistochemical staining in colon cancer tissues [38].

When pre- and post-treatment *SLFN11* expression levels were analyzed in relation to demographic and clinicopathological characteristics, only pre-treatment *SLFN11* expression was significantly positively correlated with post-treatment CEA levels, indicating a potential association between baseline *SLFN11* expression and biological characteristics reflected by post-treatment CEA levels. Post-treatment *SLFN11* expression, however, was significantly associated with disease outcome, with higher expression levels observed in patients who died during follow-up than in those who remained alive. However, observed associations did not remain significant after adjustment for multiple comparisons. Therefore, these findings should be considered exploratory and further investigated in a larger cohort of LARC patients. A previous study from our group was the first to evaluate circulating SLFN11 levels in colorectal cancer and demonstrated that higher serum SLFN11 concentrations, measured by ELISA, were associated with shorter one-year and three-year overall survival [39]. However, Kaplan–Meier analyses performed using publicly available bioinformatic platforms as well as our clinical cohort did not demonstrate a significant association between either pre- or post-treatment *SLFN11* expression and overall or disease-free survival. Nevertheless, this finding should be interpreted cautiously, as the limited number of outcome events during follow-up may have reduced the statistical power of the survival analyses to detect differences between expression groups.

When evaluating the predictive significance of *SLFN11* expression for response to nCRT, no significant association was observed in our cohort, the ROC Plotter analysis, or in seven of the eight GEO datasets analyzed. Notably, one GEO dataset demonstrated significantly lower *SLFN11* expression in patients with an incomplete pathological response compared with those who achieved a complete response. Heterogeneity among the GEO datasets may partly reflect differences in transcriptomic platforms. The only significant dataset was generated using RNA sequencing, whereas the remaining datasets were based on microarray hybridization technologies. Nevertheless, as only one dataset reached statistical significance, the overall GEO evidence does not support a consistent predictive role for *SLFN11*. These findings should be interpreted cautiously given the relatively small sample sizes of the available datasets and the limited availability of data specifically derived from patients with LARC treated with fluoropyrimidine-based neoadjuvant chemoradiotherapy. Consequently, the scarcity and heterogeneity of publicly available datasets represent an important limitation of the present study. Furthermore, only three patients in our cohort achieved a TRG1–2 response. Thus, results of the predictive potential of *SLFN11* should be interpreted with caution and considered exploratory. Tian et al. reported that high *SLFN11* expression increased the sensitivity of colorectal cancer cell lines to SN-38 by promoting apoptosis and cell cycle arrest, supporting its potential role as a predictive biomarker for irinotecan-based treatment [40]. Zoppoli et al. demonstrated that *SLFN11* expression strongly correlates with sensitivity to DNA-damaging agents, particularly topoisomerase I inhibitors, across the NCI-60 cancer cell line panel [16]. Deng et al. investigated SLFN11 protein expression in stage II–III colorectal cancer patients treated with oxaliplatin-based adjuvant chemotherapy (FOLFOX) and reported improved outcomes in patients with high SLFN11 tumor expression. Notably, this association was restricted to the *KRAS* exon 2 wild-type subgroup, suggesting that SLFN11 may serve as both a prognostic and predictive biomarker in this setting [41].

An important strength of our study is the integration of bioinformatics and experimental data obtained from paired tumor and non-tumor samples of LARC patients collected before and after nCRT. However, when analyzing the results of our study, several limitations should be considered. Although the study group was relatively small, consisting of 26 LARC patients, it had sufficient statistical power (1 − β = 0.96, effect size = 0.8, α = 0.05) to detect differences in *SLFN11* expression between tumor and non-tumor tissue before and after nCRT. In contrast, the statistical power to evaluate the prognostic significance of *SLFN11* was limited, as there were only five LARC patients who died over 80 months of follow-up. Similarly, the power to detect the predictive potential of *SLFN11* was insufficient (1 − β = 0.23, effect size = 0.8, α = 0.05), due to the small number of responders and non-responders to nCRT. In this regard, these findings should be considered exploratory and interpreted with caution. RNA degradation may be of particular concern in post-neoadjuvant chemoradiotherapy specimens and could potentially affect mRNA expression analyses. The lack of RNA integrity assessment and use of a single endogenous control represents a limitation of the current study. Future studies incorporating RNA integrity and multiple reference genes would further strengthen the reliability of gene expression analyses in clinical samples exposed to nCRT. Another limitation of this study is that *SLFN11* expression was evaluated only at the mRNA level. Since mRNA expression does not necessarily reflect protein abundance or activity, future studies incorporating protein-level validation, such as immunohistochemistry, are needed to further establish the biological and clinical significance of *SLFN11* in LARC. Overall, our study represents an initial effort toward elucidating the role of *SLFN11* in LARC. Future studies should include larger group of LARC patients, treatment-naïve normal clinical samples, analysis of *SLFN11* expression at the protein level, *in vitro* experiments using appropriate cell lines and investigation of the broader signaling pathways associated with LARC.

Although *SLFN11* did not demonstrate prognostic or predictive value in the present study, its significant treatment-induced upregulation highlights the need for further mechanistic studies of its regulation following nCRT and provides a rationale for future investigations into its potential role in treatment resistance.

## 5. Conclusions

Our integrated analyses indicate that, despite the established biological role of *SLFN11* in the DNA damage response and its treatment-induced upregulation following nCRT, no consistent evidence was found to support its prognostic or predictive value in LARC. These findings suggest that the clinical utility of *SLFN11* remains uncertain and should be evaluated in larger, adequately powered, independent cohorts incorporating both transcriptomic and protein-level analyses.

## Figures and Tables

**Table 1:** Characteristics of Gene Expression Omnibus (GEO) datasets evaluating pretreatment *SLFN11* expression in rectal cancer tissues in relation to response to neoadjuvant chemoradiotherapy.

GEO Accession No	No	R	NR	Adj. *p*	Platform	Citation	Comparison
GSE94104	40	29	11	0.999	Illumina HumanHT-12 WG-DASL V4.0 R2 expression beadchip	Alderdice et al. [26]	TRG1–2 vs. TRG3
GSE68204	59	27	32	1	Agilent-014850 Whole Human Genome Microarray 4x44K G4112F (GPL6480)	Millino et al. [27]	Responders (TRG1–2) vs. non-responders (TRG3–5)
GSE145666	6	3	3	0.318	Agilent-067406 Human CBC lncRNA + mRNA microarray V4.0	–	Good vs. poor response
GSE53781	26	10	16	0.804	CodeLink Human Whole Genome Array	Palma et al. [28]	Responders (TRG1–2) vs. non-responders (TRG3–5)
GSE242786 *	14	5	9	0.04	Illumina HiSeq 2000 (Homo sapiens)	Chong et al. [29]	Incomplete vs. complete responders
GSE46862	69	28	41	1	[HuGene-1_0-st] Affymetrix Human Gene 1.0 ST Array [transcript (gene) version]	–	Minimal (MI) and moderate (MO) response vs. near-total (NT) and total (TO) response
GSE150082	39	16	23	0.901	Agilent-026652 Whole Human Genome Microarray 4x44K v2	Sendoya et al. [30]	Good vs. poor responders: pTRG 0–1/cCR vs. pTRG 2–3/unresectable
GSE145037	31	20	11	1	Affymetrix Human Gene Expression Array	Zhang et al. [31]	Response vs. non-response

No, number of samples; R, responders; NR, non-responders; Adj., adjusted; *, Adj. *p* < 0.050.

**Table 2:** Demographic and clinicopathological characteristics of patients and their association with pre- and post-treatment *SLFN11* expression in tumor tissues.

Demographic and Clinicopathological Data		*n*	(%)	Relative Expression of *SLFN11* Before nCRT	*p*	Adj. *p*	Relative Expression of *SLFN11* After nCRT	*p*	Adj. *p*
Number of patients		26							
Sex	Male	16	61.5	0.0009 (0.0007–0.0013)	>0.999	>0.999	0.0031 (0.0024–0.0063)	0.551	0.950
	Female	10	38.5	0.0009 (0.0004–0.0019)			0.0058 (0.0025–0.0071)		
Age (years), median, range		64, 34–83		rho = −0.0230	0.911	0.911	rho = −0.1143	0.578	0.780
Tumor localization	Low rectum	13	50	0.0010 (0.0005–0.0016)	>0.999	>0.999	0.0037 (0.0027–0.0073)	0.311	0.950
	Mid rectum	13	50	0.0009 (0.0007–0.0013)			0.0032 (0.0020–0.0064)		
Clinical T stage (cT)	T3	19	73.1	0.0009 (0.0005–0.0013)	0.611	0.934	0.0032 (0.0026–0.0065)	0.866	0.950
	T4	7	26.9	0.0009 (0.0006–0.0015)			0.0055 (0.0023–0.0071)		
Clinical N stage (cN)	N1	7	26.9	0.0009 (0.0006–0.0016)	0.778	0.999	0.0032 (0.0026–0.0065)	0.910	0.950
	N2	19	73.1	0.0009 (0.0005–0.0015)			0.0037 (0.0024–0.0070)		
Clinical M stage (cM)	M0	26	100	-	-		-	-	
Prognosis	Good prognosis (T1–3/N1)	5	19.2	0.0013 (0.0007–0.0017)	0.410	0.934	0.0032 (0.0027–0.0063)	0.950	0.950
	Poor prognosis (T4 or N2)	21	80.8	0.0009 (0.0005–0.0014)			0.0037 (0.0024–0.0071)		
CRM at diagnosis	No	16	61.5	0.0009 (0.0006–0.0013)	0.856	0.999	0.0030 (0.0024–0.0069)	0.484	0.950
	Yes	10	38.5	0.0010 (0.0006–0.0017)			0.0056 (0.0028–0.0066)		
EMVI at diagnosis	No	16	61.5	0.0009 (0.0005–0.0013)	0.182	0.934	0.0031 (0.0022–0.0070)	0.623	0.950
	Yes	10	38.5	0.0013 (0.0007–0.0019)			0.0056 (0.0025–0.0064)		
CEA before therapy (IU/mL), mean ± SD		17.35 ± 46.477		rho = 0.3426	0.087	0.121	rho = 0.0907	0.660	0.780
CA 19-9 before therapy (IU/mL), mean ± SD		28.17 ± 74.784		rho = 0.3326	0.097	0.121	rho = 0.0576	0.780	0.780
Pathological T stage (pT)	T0–Tis	2	7.7	T0+1+2	0.623	0.934	T0+1+2	0.776	
	T1	1	3.8	0.0008 (0.0006–0.0014)			0.0034 (0.0023–0.0099)		
	T2	7	26.9						0.950
	T3	15	57.7	T3+4			T3+4		
	T4	1	3.8	0.0011 (0.0005–0.0015)			0.0034 (0.0024–0.0062)		
Pathological N stage (pN)	N0	16	61.5	0.0009 (0.0006–0.0011)	0.363	0.934	0.0030 (0.0023–0.0064)	0.363	
	N1	9	34.6	N1+2			N1+2		0.950
	N2	1	3.8	0.0013 (0.0005–0.0016)			0.0047 (0.0029–0.0071)		
M stage	M0	25	96.2	-	-	-	-	-	-
	M1	1	3.8	-	-		-	-	
CEA after therapy (IU/mL), mean ± SD		4.62 ± 5.872		rho = 0.4313	0.028 *	0.121	rho = 0.0787	0.702	0.780
CA 19-9 after therapy (IU/mL), mean ± SD		11.31 ± 10.054		rho = 0.3532	0.077	0.121	rho = 0.1115	0.588	0.780
Tumor regression grade (TRG) pathological (Mandard)	TRG1	2	7.7	TRG1+2	0.490	0.934	TRG1+2	0.762	0.950
	TRG2	1	3.8	0.0005 (0.0004–0.0016)			0.0032 (0.0026–0.0172)		
	TRG3	11	42.3	TRG3+4			TRG3+4		
	TRG4	12	46.2	0.0009 (0.0006–0.0015)			0.0037 (0.0024–0.0065)		
Relapse occurrence	Yes	8	30.8	0.0011 (0.0006–0.0015)	0.531	0.934	0.0046 (0.0026–0.0071)	0.644	0.950
	No	18	69.2	0.0009 (0.0005–0.0015)			0.0031 (0.0024–0.0063)		
Disease outcome	Alive	21	80.8	0.0009 (0.0006–0.0013)	0.340	0.934	0.0031 (0.0024–0.0060)	0.028 *	0.336
	Death	5	19.2	0.0015 (0.0006–0.0074)			0.0070 (0.0045–0.0444)		

Relative expression data are presented as median (interquartile range, IQR) of 2^−ΔCt^ values. The Mann–Whitney U test was applied for comparisons between groups, and Spearman’s rank correlation coefficient (rho) was used to assess associations between continuous variables. Adjusted *p*-values (Adj. *p*) were calculated using the Benjamini–Hochberg false discovery rate correction for multiple comparisons and applied separately to Mann–Whitney U test comparisons and Spearman correlation analyses. CA 19-9, carbohydrate antigen; CEA, carcinoembryonic antigen; CRM, circumferential resection margin; EMVI, extramural vascular invasion; SD, standard deviation; * *p* < 0.05.

**Table 3:** Cox-regression analysis of *SLFN11* expression before and after neoadjuvant chemoradiotherapy and survival outcomes in patients with locally advanced rectal cancer.

		Overall Survival	*p*	Disease-Free Survival	*p*
		HR (95% CI)		HR (95% CI)	
Tumor before nCRT	*SLFN11* expression (low versus high)	1.576 (0.262–9.471)	0.619	2.175 (0.543–8.714)	0.257
Tumor after nCRT	*SLFN11* expression (low versus high)	3.468 (0.360–33.437)	0.282	1.546 (0.413–5.793)	0.518

nCRT, neoadjuvant chemoradiotherapy; HR (95% CI), hazard ratio (95% confidence interval).

## Data Availability

The data supporting the findings of this study are available from the corresponding author upon reasonable request. Public release of the dataset is restricted because it contains sensitive patient information and is subject to institutional and ethical confidentiality requirements.

## Supplementary Materials

The following supporting information can be downloaded at https://www.mdpi.com/article/10.3390/biomedicines14081827/s1, Figure S1: Flowchart illustrating the sequential filtering of samples from the GDC-TCGA READ cohort used for the UCSC Xena analysis. The green box indicates selection based on genomic (RNAseq) data type, whereas the orange boxes indicate filtering based on phenotypic variables.

## Abbreviations

The following abbreviations are used in this manuscript:

95% CI	95% confidence interval
AUC	Area under the curve
CA19-9	Carbohydrate antigen 19-9
CEA	Carcinoembryonic antigen
CRC	Colorectal cancer
CRM	Circumferential resection margin
DDA	DNA-damaging agent
EMVI	Extramural vascular invasion
GDC–TCGA	Genomic Data Commons–The Cancer Genome Atlas
GEO	Gene Expression Omnibus
HDAC	Histone deacetylase
HR	Hazard ratio
IQR	Interquartile range
LARC	Locally advanced rectal cancer
nCRT	Neoadjuvant chemoradiotherapy
PARP	Poly(ADP-ribose) polymerase
READ	Rectal Adenocarcinoma project
ROC	Receiver operating characteristics
SD	Standard deviation
TNM	Tumor, node, metastasis
TRG	Tumor regression grade
UCSC	University of California, Santa Cruz
