# Supplementary material for: Integrated Bioinformatic and Experimental Analysis of SLFN11 Expression and Clinical Significance in Locally Advanced Rectal Cancer
Source: Biomedicines. 2026 Aug 13;14(8):1827. doi: 10.3390/biomedicines14081827 (PMC13509909; doi:10.3390/biomedicines14081827)
Supplement: Supplementary file 1 [file biomedicines-14-01827-s001.zip › biomedicines-4457910-supplementary.pdf]

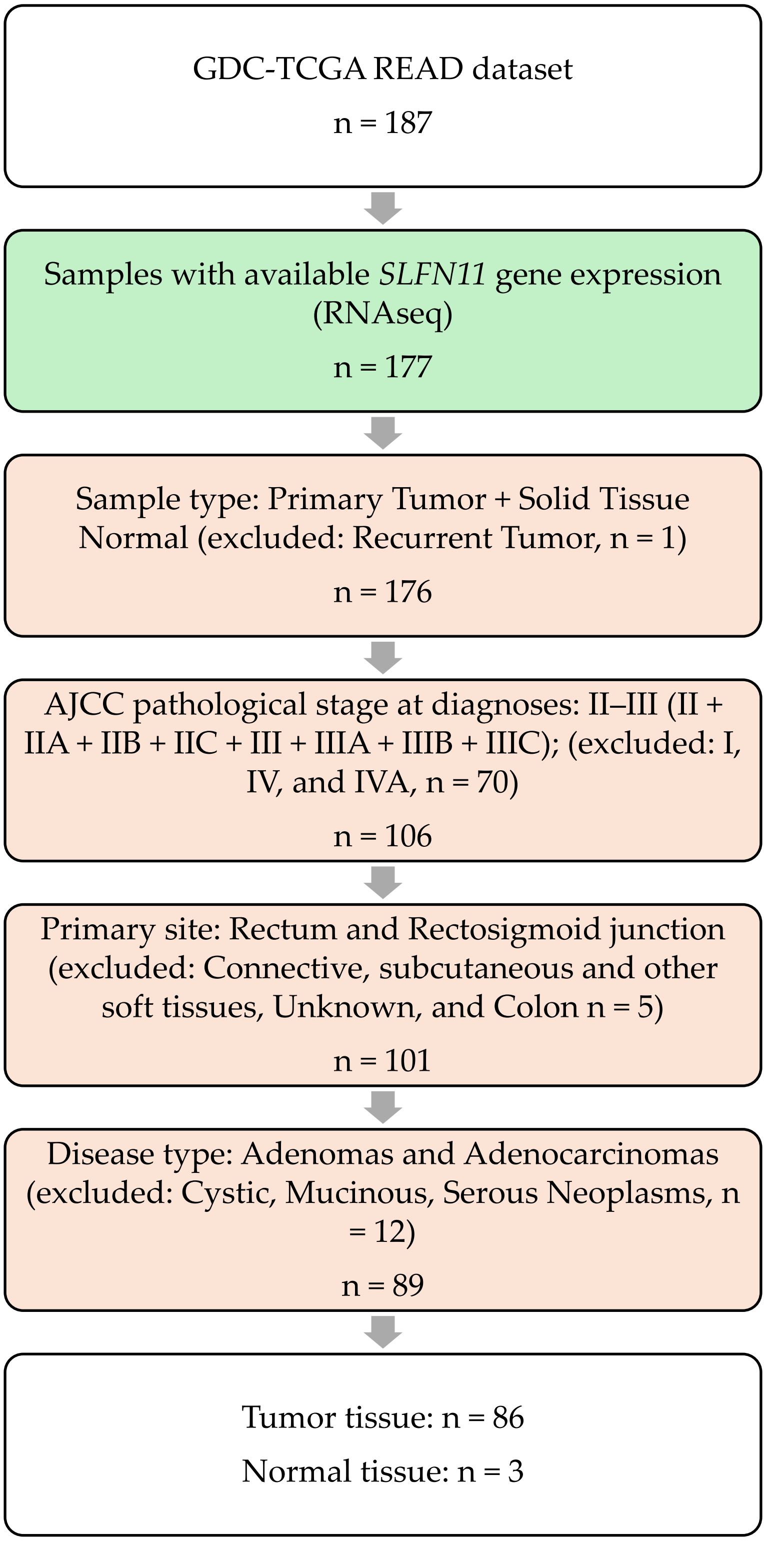

Supplement Figure S1. Flowchart illustrating the sequential filtering of samples from the GDC-TCGA READ cohort used for the UCSC Xena analysis. The green box indicates selection based on genomic (RNAseq) data type, whereas the orange boxes indicate filtering based on phenotypic variables.
